# Differential regulation of Cav2.2 channel exon 37 variants by alternatively spliced μ-opioid receptors

**DOI:** 10.1186/s13041-019-0524-6

**Published:** 2019-11-27

**Authors:** Maria A. Gandini, Ivana A. Souza, Dvij Raval, Jin Xu, Ying-Xian Pan, Gerald W. Zamponi

**Affiliations:** 1 Department of Physiology and Pharmacology, Alberta Children’s Hospital Research Institute, Hotchkiss Brain Institute, Cumming School of Medicine, University of Calgary, Calgary, AB Canada; 2 Department of Neurology and the Molecular Pharmacology Program, Memorial Sloan-Kettering Cancer Center, New York, NY USA

**Keywords:** N-type, Calcium channel, Cav2.2, Opioid receptor, Splicing, G proteins

## Abstract

We have examined the regulation of mutually exclusive Cav2.2 exon 37a and b variants by the mouse μ-opioid receptor (mMOR) C-terminal splice variants 1, 1C and 1O in tsA-201 cells. Electrophysiological analyses revealed that both channel isoforms exhibit DAMGO-induced voltage-dependent (Gβγ-mediated) inhibition and its recovery by voltage pre-pulses, as well as a voltage-independent component. However, the two channel isoforms differ in their relative extent of voltage-dependent and independent inhibition, with Cav2.2-37b showing significantly more voltage-dependent inhibition upon activation of the three mMOR receptors studied. In addition, coexpression of either mMOR1 or mMOR1C results in an agonist-independent reduction in the peak current density of Cav2.2-37a channels, whereas the peak current density of Cav2.2-37b does not appear to be affected. Interestingly, this decrease is not due to an effect on channel expression at the plasma membrane, as demonstrated by biotinylation experiments. We further examined the mechanism underlying the agonist-independent modulation of Cav2.2-37a by mMOR1C. Incubation of cells with pertussis toxin did not affect the mMOR1C mediated inhibition of Cav2.2-37a currents, indicating a lack of involvement of Gi/o signaling. However, when a Src tyrosine kinase inhibitor was applied, the effect of mMOR1C was lost. Moreover, when we recorded currents using a Cav2.2-37a mutant in which tyrosine 1747 was replaced with phenylalanine (Y1747F), the agonist independent effects of mMOR1C were abolished. Altogether our findings show that Cav2.2-37a and Cav2.2-37b isoforms are subject to differential regulation by C-terminal splice variants of mMORs, and that constitutive mMOR1C activity and downstream tyrosine kinase activity exert a selective inhibition of the Cav2.2-37a splice variant, an N-type channel isoform that is highly enriched in nociceptors. Our study provides new insights into the roles of the MOR full-length C-terminal variants in modulating Cav2.2 channel isoform activities.

## Introduction

Voltage-gated calcium channels trigger depolarization-mediated calcium influx into electrically excitable cells in nerve, muscle and heart (for review see [1]). The nervous system expresses multiple types of calcium channels encompassing three distinct families (Cav1, Cav2 and Cav3) with specialized functional roles. Calcium entry via voltage gated calcium channels can be further tuned by a number of factors, including phosphorylation [2–5], post-translational modifications such as glycosylation [6–8] and ubiquitination [9–12], interactions with adaptor proteins [13–17], and association with synaptic proteins [18] (for review see [19]). These examples highlight the vast spectrum of regulatory mechanisms that fine tune calcium entry into neurons.

**Fig. 1:**
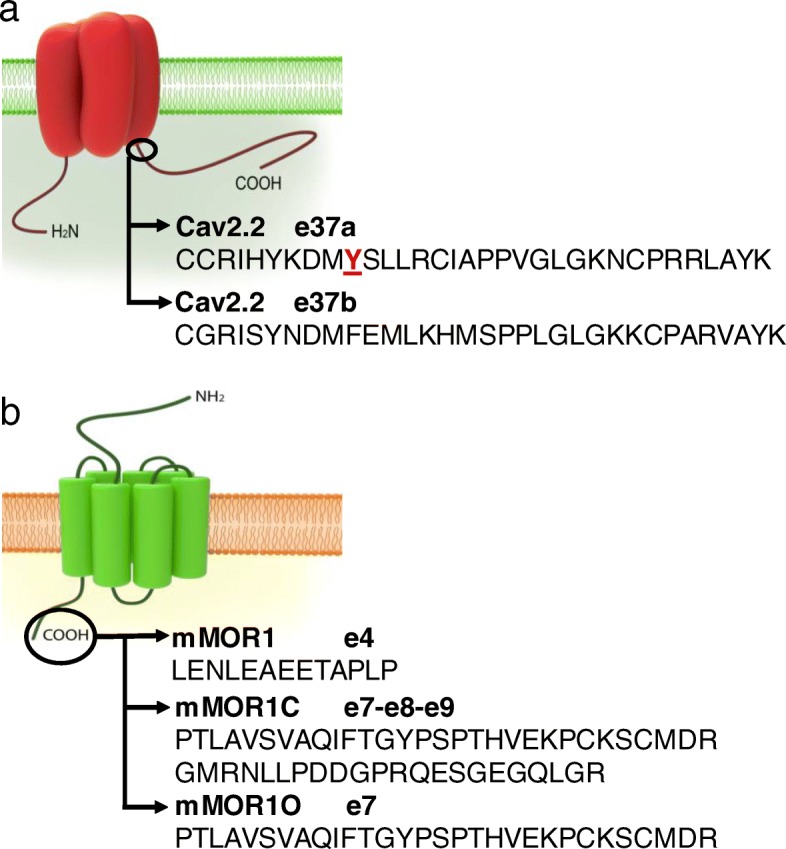
Schematic representation of Cav2.2 channels and mMORs. **a** The Cav2.2 proximal C-terminus is in part encoded by the mutually exclusive exons 37a and 37b. The illustration shows the amino acids encoded by each exon. Shown in red is the tyrosine that was mutated for the experiments in Fig. 6. **b** MOR full-length distal C-terminal variants are produced by alternative splicing and amino acid sequences encoded by exon 4, 7 and 7–8-9 are depicted

Each of the known calcium channel isoforms is known to undergo alternative mRNA splicing [1, 20, 21], thus adding to functional diversity of this channel family. The physiological impact of alternate splicing of calcium channels is exemplified by studies on Cav1.2 L-type channels showing that inclusion of exon33 contributes to heart failure and cardiac arrhythmias [22, 23], by the notion that a mutation in Cav3.2 linked to epilepsy functionally manifests itself only in variants containing exon 25 [24] and by findings with Cav2.2 N-type channels where alternative splicing of exon 37 was shown to affect transmission of peripheral pain signals [25–27]. In Cav2.2, there are two mutually exclusive variants of exon 37 (exon37a and exon 37 b) whose inclusion/exclusion leads to a difference in 14 amino acid residues within the C-terminal region of the Cav2.2 α1 subunit (Fig. 1a), with the exon 37a variant being highly enriched in nociceptors [25]. These sequence differences have important effects on the ability of the channel to respond to modulation by G protein βγ subunits. Indeed, it has been shown G protein inhibition of Cav2.2 channels triggered by activation of μ-opioid receptors (MORs) is altered in channels containing exon 37a [28, 29], such that there is an increase in tyrosine kinase-mediated voltage-independent inhibition and less classical voltage-dependent modulation that involves direct Gβγ interactions with the channel [30–33].

MOR full-length C-terminal splice variants, produced by 3′ alternative pre-mRNA splicing of the *OPRM1* gene, have been reported in different species. These variants have identical receptor structures, but contain a unique intracellular C-terminal tail sequence, and are known to exhibit different cellular and regional distributions [34–36]. The original mMOR1 includes a C-terminal tail sequence encoded by exon 4 with 12 amino acids. Both mMOR1C and mMOR1O have an alternative C-terminal tail encoded by exon 7a with a unique 30 amino acid sequence, while mMOR1C contains additional exons 8/9 with an extra 22 amino acids (Fig. 1b). These variants exhibit different signalling bias and differentially contribute to various morphine actions including morphine tolerance, physical dependence, reward behavior and locomotor activity profile without affecting morphine analgesia [37]. We thus wondered whether these receptor variants may couple differentially to Cav2.2 channels, and if so, whether this may occur in a Cav2.2 splice isoform specific manner. Here, we report that different combinations of mMOR1, mMOR1C and mMOR1O and rat Cav2.2 exon 37 isoforms exhibit distinct voltage dependent and independent modulation.

## Materials and methods

### cDNA transfection

tsA-201 cells were transfected with 3 μg of each plasmid encoding Cav2.2α1 (WT or Y1747F mutant), Cavβ1 and Cavα2δ-1, respectively, in the presence of empty vector, or mMOR1, mMOR1C or mMOR1O using the calcium phosphate method as described previously [38]. In addition, 0.5 μg of cDNA encoding green fluorescent protein was added to the transfection mixture to identify and select transfected cells. Cells used for electrophysiology experiments were moved to 30 °C after transfection, whereas those used for Western blotting were maintained at 37 °C.

### Electrophysiology recordings

Whole cell patch-clamp recordings were performed at room temperature (22–24 °C). Currents were recorded using an Axopatch 200B amplifier linked to a computer with pCLAMP9.2 software. The external recording solution contained (in mM): 2 CaCl_2_, 137 CsCl, 1 MgCl_2_, 10 HEPES, 10 glucose (pH 7.4 adjusted with CsOH). The pipette solution contained (in mM): 130 CsCl, 2.5 MgCl2, 10 HEPES, 10 EGTA, 3 ATP, 0.5 GTP (pH 7.4 adjusted with CsOH). I peak was obtained by dividing the peak current by the whole cell capacitance. Current-voltage relations were fitted using the Boltzmann equation to obtain the half activation voltage. Time constants of activation were obtained by mono-exponential fits to the late rising phase of the current. The effects of receptor coexpression or pharmacological treatments on Cav2.2 current densities were always assessed in the same batch of cells. G protein modulation induced by μ-opioid receptor activation was assessed as described in the results section. Cells expressing Cav2.2-37a and mMOR1C were incubated overnight with 500 ng/ml of PTX (Tocris 3097) or with 2 μM of Src inhibitor for 4 h (PP1, Millipore 567,809).

### Cell surface biotinylation

Cell surface biotinylation experiments were performed as previously described [38]. Briefly, surface proteins from transfected cells were biotinylated for 1 h on ice with 1 mg/ml of EZ- Link Sulfo-NHS-SS-Biotin (Thermo Scientific, 21,331). The reaction was quenched with 100 mM glycine for 15 min, and cells were lysed in modified RIPA buffer (in mM: 50 Tris, 150 NaCl, 5 EDTA, 1% Triton X-100, 1% NP-40, 0.2% SDS, pH 7.4) for 45 min. Two mg of lysates were incubated with 100 μl of Neutravidin beads (Thermo Scientific 29,200) for 1.5 h at 4 °C. Beads were washed and proteins eluted with 2× Laemmli sample buffer. Biotinylated proteins and lysates were resolved by SDS-PAGE and analyzed by western blot using the antibodies anti-Cav2.2 (1:500, Alomone ACC-002) and anti-Na/K ATPase (1:5000, Abcam AB 7671).

### Statistical analysis

All error bars reflect standard errors. All data were analyzed for normality using D’Agostino and Pearson tests. Normal data were statistically analyzed using Student’s t-tests or One-way Analysis of variance (ANOVA) for multiple comparisons. Pre-pulse facilitation was analyzed using a Wilcoxon matched-pairs test. Non normally distributed data were analyzed via a Mann-Whitney test, or a Kruskal-Wallis test for multiple comparisons. Significance was set at 0.05. Asterisks denote significance as follows: * *p* < 0.05, ** *p* < 0.01, ***p < 0.001. Unless stated otherwise, data are presented as means plus standard errors.

## Results and discussion

### Cav2.2-37a channels are subject to agonist-independent modulation by mMOR variants

**Fig. 2:**
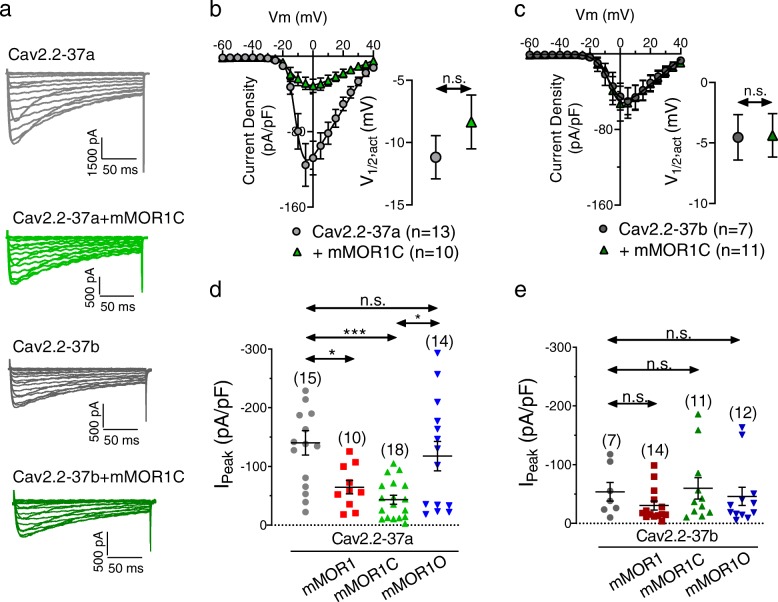
Peak current densities (I peak) of Cav2.2e37a and Cav2.2e37b channels coexpressed with mMOR1, mMOR1C or mMOR1O.**a** Representative whole cell current traces recorded in response to depolarizing steps from − 60 mV to + 40 mV from a holding potential of − 80 mV from tsA-201 cells expressing Cav2.2-37a/Cavβ1/Cavα2δ-1 or Cav2.2-37b /Cavβ1/Cavα2δ-1 channels plus/minus mMOR1C. **b** Average current density-voltage relationships for cells expressing Cav2.2-37a channels with or without mMOR1C. Inset: Corresponding mean half activation potentials. **c** Average current density-voltage relationships for cells expressing Cav2.2-37b channels with or without mMOR1C. Inset: Corresponding mean half activation potentials. **d** Average peak current density for whole cell calcium currents recorded from cells expressing Cav2.2e37a/Cavβ1/Cavα2δ-1 in the presence of mMOR1, mMOR1C and mMOR1O. **e** Average peak current density recorded from tsA-201 cells expressing Cav2.2e37b/Cavβ1/Cavα2δ-1 in the presence of mMOR1, mMOR1C or mMOR1O. The numbers in parentheses represent the number of cells recorded. n.s. – not significant, asterisks denote significance at the *0.05 and ***0.001 levels (**d** – ANOVA; **e -** Kruskal-Wallis test)

**Fig. 3:**
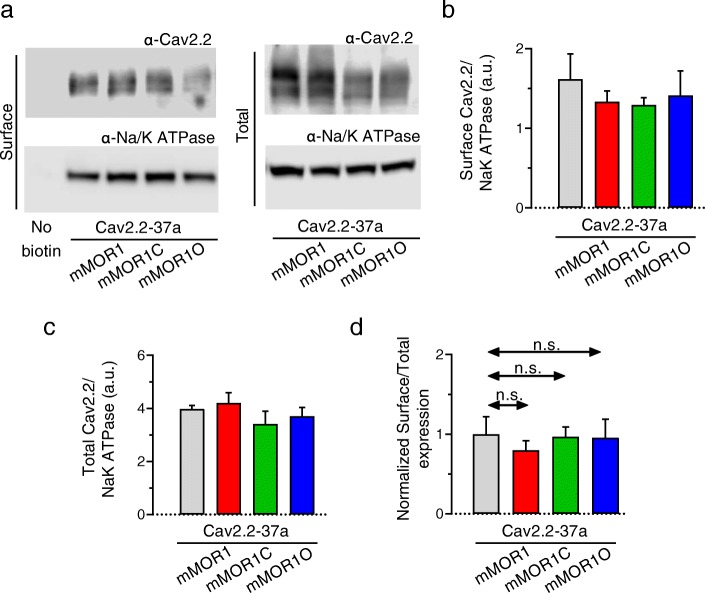
Biotinylation of Cav2.2e37a/Cavβ1/Cavα2δ-1 in the presence of mMOR1, mMOR1C and mMOR1O.Biotinylated cell surface proteins were isolated and normalized to Na/K-ATPase levels. **a** Representative blot of Cav2.2-37a surface and total expression (top blots) and Na/K-ATPase surface and total expression (bottom blots). **b** Quantification of plasma membrane Cav2.2-37a/ Cavβ1/Cavα2δ-1 channel expression in the absence and the presence of mMOR1, mMOR1C or mMOR1O (normalized by Na/K-ATPase cell surface expression). **c** Quantification of total Cav2.2-37a/Cavβ1/Cavα2δ-1 expression in the absence or the presence of mMOR1, mMOR1C or mMOR1O (normalized by total Na/K-ATPase expression). **d** Normalized surface to total expression of Cav2.2-37a channels. Data are from 4 independent experiments. n.s. – not significant (Kruskal-Wallis test)

We have previously reported that coexpression of Cav2.2 with members of the extended opioid receptor family can produce agonist-independent inhibitory effects [39, 40]. We thus first examined current peak densities of rat Cav2.2-37a and Cav2.2-37b variants (coexpressed with rat Cavβ1b and rat Cavα2δ1 subunits) in the absence and presence of mMOR1, mMOR1C or mMOR1O in tsA-201 cells (Fig. 2).Cav2.2-37a channels exhibited larger whole cell current densities than Cav2.2-37b, in agreement with previous findings [25] (Fig. 2a, b). Coexpression with mMOR1C produced a significant decrease in Cav2.2-37a average current density (Fig. 2b), a slight slowing of the time constant for activation at some of the test potentials (not shown), but no change in half-activation potential (Fig. 2b inset). In contrast, there was no change in current density when Cav2.2-37b was coexpressed with mMOR1C (Fig. 2c). Figure 2d and e examine the effects of other MOR variants on the two Cav2.2 isoforms. While current densities of Cav2.2-37b were largely insensitive to coexpression of MOR variants, Cav2.2-37a channels exhibited significantly lower whole cell peak current densities in the presence of mMOR1 (Cav2.2-37a: − 140.2 ± 20.7 pA/pF vs Cav2.2-37a + mMOR1: –64.7 ± 11.6 pA/pF; *p* < 0.05) and this effect was more pronounced when the channel was coexpressed with mMOR1C (− 43.3 ± 7.6 pA/pF; *p* < 0.001) (Fig. 2d**,** Additional file 1: Figure S1). By contrast, coexpression of mMOR1O was largely innocuous (− 117.6 ± 25.1 pA/pF). These data indicate that Cav2.2-37a channels are either functionally inhibited by mMOR1 and mMOR1C, or that these receptors might affect trafficking of the channels to the cell surface. To discriminate among the alternatives, we performed cell surface biotinylation experiments with Cav2.2-37a. As shown in Fig. 3, none of the receptor isoforms affected cell surface expression of the channels (Fig. 3b and d) or their total expression (Fig. 3c) indicating that mMOR1 and mMOR1C functionally inhibit Cav2.2 channels even in the absence of agonist, rather than affecting channel cell surface expression.

### Exon 37 Cav2.2 variants exhibit differential degrees of DAMGO-induced voltage-dependent and independent modulation

**Fig. 4:**
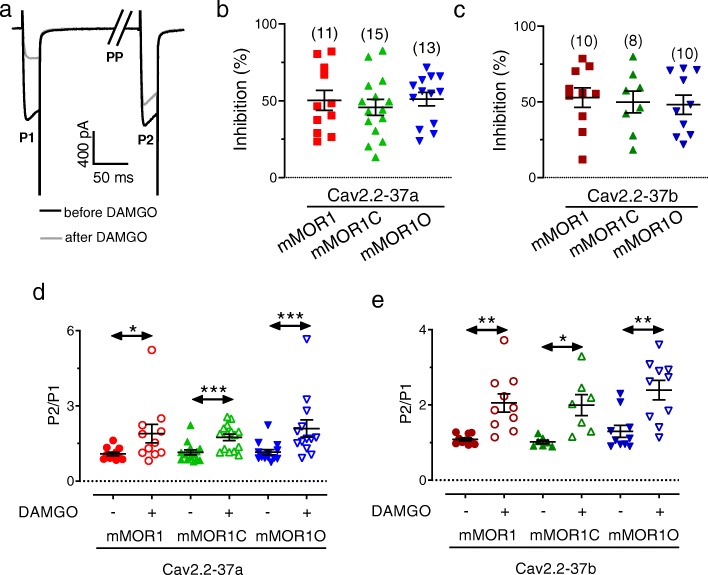
G protein modulation of Cav2.2-37a and Cav2.2-37b channels following activation of mMOR1, mMOR1C and mMOR1O. **a** Representative set of Cav2.2-37a currents in the presence of mMOR1C, recorded before or after the application of 10 μM DAMGO. As outlined in the Results section, P1 represents the first current in each trace evoked by a test depolarization to + 10 mV, P2 is the second inward current in a given trace evoked by a 10 mV test depolarization (P2) preceded by a strong depolarizing prepulse (PP, note that the pre-pulse-evoked outward current is not shown in the figure). Relief of Gβγ modulation by the pre-pulse is observed by the increase in current amplitude seen during P2 in the presence of DAMGO. **b** Percentage of peak current inhibition (during P1) of Cav2.2e-37a currents after application of 10 μM DAMGO. **c** Percentage of peak current inhibition (during P1) of Cav2.2e-37b currents after application of 10 μM DAMGO. **d** Voltage dependent pre-pulse facilitation measured in the presence of DAMGO in tsA-201 cells expressing Cav2.2-37a channels with mMOR1, mMOR1C or mMOR1O. The data points reflect the current evoked by test pulse P2 normalized to the current evoked by test pulse P1. **e** Voltage dependent pre-pulse facilitation measured in the presence of DAMGO in tsA-201 cells expressing Cav2.2-37b channels with mMOR1, mMOR1C or mMOR1O. The data points reflect the current evoked by the test pulse P2 normalized to the current evoked by test pulse P1. The number of cells recorded are indicated in parentheses, asterisks denote significance at the *0.05, **0.01, and ***0.001 levels (unpaired Wilcoxon test)

**Fig. 5:**
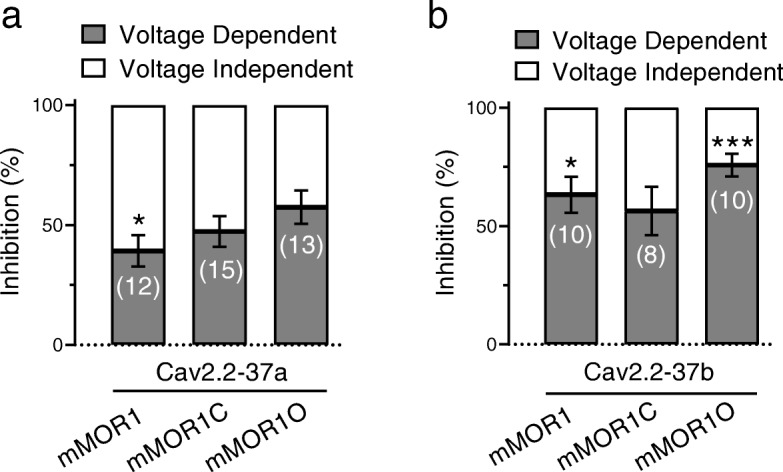
Voltage dependent and voltage independent components of DAMGO-induced modulation of Cav2.2 variants by the various MORs. **a** Voltage-dependent and independent inhibition of Cav2.2-37a channels coexpressed with mMOR1, mMOR1C and mMOR1O. **b** Voltage-dependent and independent inhibition of Cav2.2-37b channels coexpressed with mMOR1, mMOR1C and mMOR1O. The number of cells recorded are indicated in parentheses, asterisks denote significance at the *0.05 and ***0.001 levels (t-test) between voltage-dependent and voltage-independent modulation for each receptor-channel combination

Next we examined the agonist-induced (10 μM DAMGO) functional coupling between mMORs and Cav2.2 variants. For this purpose, a dual pulse protocol was applied to test for putative voltage-dependent (i.e., Gβγ- mediated) and voltage-independent components as described by us previously [41]. In brief, from a holding potential of − 80 mV, a 25 ms test depolarization (P1) to + 10 mV was applied to ascertain current amplitude. The cells were then repolarized to − 80 mV for 500 ms to recover the channels from any voltage-dependent inactivation induced by the test pulse. Then the cell was depolarized strongly to + 100 mV for 50 ms (PP) to dissociate any bound Gβγ subunits from the channel [42] which was followed by a 5 ms step to − 80 mV prior to application of a second + 10 mV test pulse (P2) (Fig. 4a). This allowed us to extract the following parameters: 1) any tonic Gβγ modulation is determined by the ratio of current amplitudes during P2 and P1 in the absence of agonist; 2) total agonist-induced G protein inhibition reflected by the DAMGO-induced reduction in Cav2.2 current amplitude during P1; 3) total voltage-dependent Gβγ modulation is ascertained by calculating the ratio of current amplitudes during P2 and P1 in the presence of DAMGO; and 4) voltage-independent agonist-mediated inhibition is reflected by the ratio of current amplitudes during P2 in the presence and the absence of DAMGO (i.e., the voltage pre-pulse removes all voltage-dependent modulation during P2, and hence all remaining DAMGO-mediated inhibition is voltage-independent). Fig. 4b-e reveals the result of this analysis. Both channel variants exhibited a similar degree of total DAMGO-mediated inhibition of ~ 50% percent irrespective of mMOR splice isoform (Fig. 4b and c). There was little if any tonic Gβγ modulation of the channels (as determined by the current amplitude ratio P2/P1 in the absence of agonist) with the exception of Cav2.2-37a channels coexpressed with mMOR1C and mMOR1O and Cav2.2-37b coexpressed with mMOR1O where significant agonist-independent pre-pulse facilitation could be observed (mean values for P2/P1 Cav2.2-37a: +mMOR1C: 1.15, +mMOR1O: 1.15 or Cav2.2-37b + mMOR1O: 1.36; Fig. 4d and e**)**. In every case, there was a strong pre-pulse relief of DAMGO inhibition consistent with agonist mediated activation of Gβγ modulation of the channels (Fig. 4d and e). A more detailed analysis of voltage-dependent and voltage independent components of agonist-induced modulation revealed that voltage independent modulation followed a pattern of mMOR1 > mMOR1C > mMOR1O for Cav2.2-37a, and mMOR1C > mMOR1 > mMOR1O for Cav2.2-37b. Hence, Cav2.2–37 inhibition by mMOR1 exhibited a large degree of voltage-independent modulation, whereas there was a predominantly voltage-dependent effect in the Cav2.2-37b + MOR1O combination (Fig. 5a and b). Altogether these data are indicative of differences in DAMGO mediated coupling between the various mMOR isoforms to the two exon 37 Cav2.2 variants.

### Agonist-independent modulation of Cav2.2-37a by mMOR1C involves tyrosine kinases

**Fig. 6:**
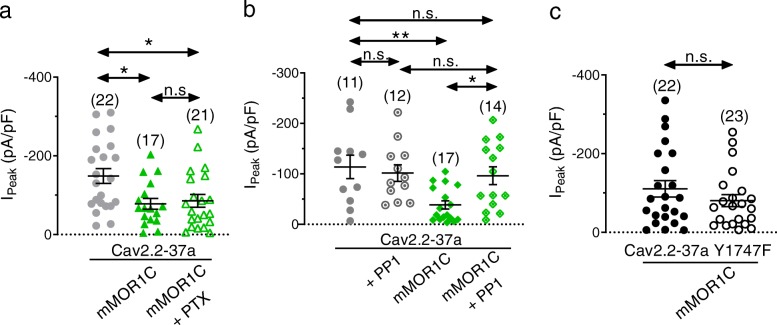
Inhibition of Src and Cav2.2-37a Y1747F abolish the effect of mMOR1C on Cav2.2-37a peak current density. **a** Peak current density of Cav2.2-37a channels treated overnight with vehicle (0.1% water or 500 ng/ml of PTX overnight. **b** Peak current density of Cav2.2-37a channels treated for 4 h with vehicle (0.004% DMSO) or 2 μM of the Src inhibitor PP1. **c** Peak current density of the Cav2.2-37a Y1747F mutant in the absence and the presence of mMOR1C. The number of cells recorded is indicated in parentheses, asterisks denote significance at the *0.05 and **0.01 levels (**a** and **b** – ANOVA, **c** - Mann-Whitney test)

It is interesting to note that although there was a significant agonist-independent pre-pulse effect on Cav2.2-37a channels particularly when coexpressed with mMOR1C, this tonic Gβγ modulation is insufficient to account for the massive agonist-independent reduction in whole cell current density observed in Fig. 2a and d. Along these lines, there appeared to be no pre-pulse mediated current enhancement of Cav2.2 + mMOR1 in the absence of agonist **(**Fig. 4c**)**, and yet the mere presence of the receptor resulted in ~ 50% smaller current densities. Given that cell surface expression was unaffected (Fig. 3), these observations indicate that Cav2.2-37a channels are inhibited in an agonist-independent and non Gβγ mediated manner by mMOR1 and mMOR1C variants. To test this hypothesis, we performed additional recordings of Cav2.2-37a channels with mMOR1C after incubation of cells with pertussis toxin (PTX) overnight. Figure 6a shows that PTX does not reverse the effects of mMOR1C on Cav2.2-37a peak current density, indicating that Gi/o signaling is not involved. It has been reported previously that Cav2.2-37a channels can be regulated by tyrosine kinases [28]. To determine whether the agonist-independent modulation involves a receptor mediated activation of such a kinase pathway, we incubated the cells for 4 h with the Src inhibitor PP1 (2 μM). As shown in Fig. 6b**,** PP1 treatment abolished the effects of mMOR1C coexpression on Cav2.2-37a current density (Cav2.2-37a + PP1: –101.6 ± 16.36 pA/pF, Cav2.2-37a + mMOR1C + PP1: –96.24 ± 17.63 pA/pF; n.s.:; n.s.) indicating that tyrosine kinase phosphorylation is needed for this type of regulation. To confirm this, we used a Cav2.2-37a mutant in which tyrosine 1747 was replaced by phenylalanine (Y1747F), a residue that was previously implicated in being a target for Src kinase [28]. As shown in Fig. 6c, current densities of this mutant were resistant to coexpression of mMOR1C (Fig. 6c), demonstrating that tyrosine 1747 is a key determinant of the agonist-independent effects of mMOR1C on Cav2.2-37a peak current density.

## Discussion

MORs are expressed in both the afferent pain pathway and within the brain’s pain matrix and are the target of nearly all the opioids used to treat pain [43]. In the afferent pain pathway, the activation of MORs leads to the activation of GIRK channels and the inhibition of N-type channel mediated calcium entry, thus attenuating neuronal excitability and synaptic communication in the spinal dorsal horn [44]. Opioid receptors are G protein coupled receptors that feature 7 transmembrane helices and a large cytoplasmic C-terminal region [45, 46]. The C-terminus is important for G protein activation, as well as phosphorylation-dependent recruitment of β-arrestins [47, 48]. Hence, alternative splicing of the MOR C-terminal region has the propensity to alter MOR signaling, as demonstrated clearly by Xu and colleagues [37] that various MOR full-length C-terminal variants can have differences in biased signaling between β- arrestin recruitment and G protein activation. Our data suggest novel roles of MOR full-length C-terminal variants in the regulation of Cav2.2 channel isoform activity. We show that three C-terminal variants, mMOR1, mMOR1C and mMOR1O, can differentially regulate Cav2.2-37a activity at the basal level (i.e., agonist-independent state), with a limited effect on Cav2.2-37b. The mere coexpression of mMOR1C and mMOR1 significantly suppressed Cav2.2-37a current densities. This regulation seems unrelated to the expression level of Cav2.2-37a. Rather, the constitutive receptor variant activities, particularly those of mMOR1C, produced a form of tonic voltage-independent modulation that appears to preferentially target exon 37a containing channels. Furthermore, the voltage-independent inhibition in absence of receptor agonist could be attributed to selective tyrosine kinase phosphorylation of Cav2.2-37a by Src acting on its Y1747F residue. This effect appears to be due to constitutive receptor activity, since the effects of MOR1C were abolished upon incubation with the Src inhibitor PP1. The notion that the effects of MOR1C coexpression were insensitive to PTX indicates that such constitutive activity does not involve Gi/o signaling. Although we do not discount the possibility that other phosphorylation sites on the channel might also play a role, the Y1747F mutant was entirely resistant to the agonist-independent effects of mMOR1C on peak current density, thus suggesting that the agonist independent receptor signaling affects current densities by primarily targeting residue Y1747. Interestingly, Raingo et al. [28] showed that the Y1747F mutant exhibited a drastic reduction in agonist-induced voltage-independent inhibition of Cav2.2-37a, resulting in a regulation that more closely approximates that of the Cav2.2-37b isoform, suggesting that our observations presented in Fig. 5 (i.e., largest voltage-independent modulation with the mMOR1/Cav2.2-37a combination) may also be due to tyrosine kinase regulation. Given that Cav2.2-37a also showed agonist mediated voltage-independent modulation despite being constitutively modulated by Src would then suggest an additional Src/Y1747 independent component of voltage-independent modulation in the presence of DAMGO. This is supported by the notion that Cav2.2-37b channels still exhibit agonist-mediated voltage-independent modulation despite the absence of a C-terminal Src site. This indicates that this type of inhibition involves additional mechanisms common to both splice isoforms which require further investigation.

mMOR1, mMOR1C and mMOR1O share same receptor sequences except for the differences in their intracellular C-terminal tails. mMOR1 has a C-terminal tail that contains 12 amino acids encoded by exon 4, while both mMOR-1C and mMOR1O have 30 amino acids encoded by exon 7a with an additional 22 amino acids encoded by exons 8/9 in mMOR1C. The agonist-independent inhibition of Cav2.2-37a by mMOR1C and mMOR1, but not by mMOR1O, raises interesting questions how these different C-terminal sequences influence Cav2.2-37a channel activity. We also demonstrated that all three receptor variants can modulate DAMGO-induced changes in pre-pulse facilitation ratio for both Cav2.2-37a and Cav2.2-37b. Exon-4 associated mMOR1 and exon 7-associated mMOR1C and mMOR-1O are differentially expressed in various brain regions at both mRNA and protein levels [34, 35, 37]. This suggests a potentially specific function of each individual receptor variant in modulating Cav2.2 isoforms in various brain regions.

Even though there were no significant differences in pre-pulse facilitation among three variants in the presence of DAMGO, a more detailed analysis reveals differences in the relative proportion of voltage-dependent and voltage-independent modulation of the Cav2.2 isoforms by the different mMOR variants, further highlighting the importance of the C-terminal sequences in Cav2.2 activity. A distinct opioid receptor mediated modulation of the two Cav2.2 exon 37 variants is consistent with earlier findings of Raingo et al. [28]. These authors showed that both exon 37 variants underwent a similar degree of total agonist mediated current inhibition, but the relative proportion of voltage-independent to voltage dependent modulation was larger in the Cav2.2-37a variant, and this fits with the data presented in Fig. 5.

It is now well established that MORs couple differentially to different members of the Cav2 channel family [49, 50], which is consistent with observations that these channels are differentially modulated by Gβγ subunits [51–53]. Moreover, different members of the extended opioid receptor family including NOP receptors types produce differential effects on Cav2 calcium channels and this is further modulated by receptor heteromerization [40, 54]. As we show here, this functional diversity of opioid receptor family signaling to various calcium channels is further expanded by MOR and Cav2.2 splice variation. While we have focused on only a subset of known mMOR splice variants, mouse, human and rat MORs have a much richer complement of possible variants [36] and it will be interesting to examine how these different isoforms of the MOR receptor affect N-type channel function and perhaps trafficking. Suffice it to say, our findings along with those published in previous literature highlight the importance of selecting the correct variants for expression studies when attempting to correlate findings between recombinant and native systems.

## Supplementary information

**Additional file 1: Figure S1.** Effect of mMOR1 and mMOR1O coexpression on Cav2.2e37a channel amplitudes. **a** Representative whole cell current traces recorded in response to depolarizing steps from a holding potential of − 80 mV from tsA-201 cells expressing Cav2.2-37a/Cavβ1/Cavα2δ-1 in the presence of mMOR1 or mMOR1O. **b** Average current density-voltage relationships for cells expressing Cav2.2-37a channels with or without mMOR1 or mMOR1O. The data obtained in the absence of receptors are the same as those shown in Fig. 2b

## Data Availability

The data used in our study are available from the authors on reasonable request.

## References

[1] Zamponi GW, Striessnig J, Koschak A, Dolphin AC (2015). The Physiology, Pathology, and Pharmacology of Voltage-Gated Calcium Channels and Their Future Therapeutic Potential. Pharmacol Rev..

[2] Iftinca MC, Zamponi GW (2009). Regulation of neuronal T-type calcium channels. Trends Pharmacol Sci..

[3] Blesneac I, Chemin J, Bidaud I, Huc-Brandt S, Vandermoere F, Lory P (2015). Phosphorylation of the Cav3.2 T-type calcium channel directly regulates its gating properties. Proc Natl Acad Sci U S A..

[4] Raifman TK, Kumar P, Haase H, Klussmann E, Dascal N, Weiss S (2017). Protein kinase C enhances plasma membrane expression of cardiac L-type calcium channel, CaV1.2. Channels (Austin)..

[5] Schneider T, Alpdogan S, Hescheler J, Neumaier F (2018). In vitro and in vivo phosphorylation of the Cav2.3 voltage-gated R-type calcium channel. Channels (Austin).

[6] Weiss N, Black SA, Bladen C, Chen L, Zamponi GW (2013). Surface expression and function of Cav3.2 T-type calcium channels are controlled by asparagine-linked glycosylation. Pflugers Arch.

[7] Orestes P, Osuru HP, McIntire WE, Jacus MO, Salajegheh R, Jagodic MM, Choe W, Lee J, Lee SS, Rose KE, Poiro N, Digruccio MR, Krishnan K, Covey DF, Lee JH, Barrett PQ, Jevtovic-Todorovic V, Todorovic SM (2013). Reversal of neuropathic pain in diabetes by targeting glycosylation of Ca(V)3.2 T-type calcium channels. Diabetes..

[8] Liu Y, Wang P, Ma F, Zheng M, Liu G, Kume S, Kurokawa T, Ono K (2019). Asparagine-linked glycosylation modifies voltage-dependent gating properties of CaV3.1-T-type Ca(2+) channel. J Physiol Sci.

[9] Waithe D, Ferron L, Page KM, Chaggar K, Dolphin AC (2011). Beta-subunits promote the expression of Ca(V)2.2 channels by reducing their proteasomal degradation. J Biol Chem..

[10] Altier C, Garcia-Caballero A, Simms B, You H, Chen L, Walcher J, Tedford HW, Hermosilla T, Zamponi GW (2011). The Cavbeta subunit prevents RFP2-mediated ubiquitination and proteasomal degradation of L-type channels. Nat Neurosci..

[11] Marangoudakis S, Andrade A, Helton TD, Denome S, Castiglioni AJ, Lipscombe D (2012). Differential ubiquitination and proteasome regulation of Ca(V)2.2 N-type channel splice isoforms. J Neurosci..

[12] Garcia-Caballero A, Gadotti VM, Stemkowski P, Weiss N, Souza IA, Hodgkinson V, Bladen C, Chen L, Hamid J, Pizzoccaro A, Deage M, Francois A, Bourinet E, Zamponi GW (2014). The deubiquitinating enzyme USP5 modulates neuropathic and inflammatory pain by enhancing Cav3.2 channel activity. Neuron..

[13] Kiyonaka S, Wakamori M, Miki T, Uriu Y, Nonaka M, Bito H, Beedle AM, Mori E, Hara Y, De Waard M, Kanagawa M, Itakura M, Takahashi M, Campbell KP, Mori Y (2007). RIM1 confers sustained activity and neurotransmitter vesicle anchoring to presynaptic Ca2+ channels. Nat Neurosci..

[14] Polster A, Perni S, Bichraoui H, Beam KG (2015). Stac adaptor proteins regulate trafficking and function of muscle and neuronal L-type Ca2+ channels. Proc Natl Acad Sci U S A..

[15] Grauel MK, Maglione M, Reddy-Alla S, Willmes CG, Brockmann MM, Trimbuch T, Rosenmund T, Pangalos M, Vardar G, Stumpf A, Walter AM, Rost BR, Eickholt BJ, Haucke V, Schmitz D, Sigrist SJ, Rosenmund C (2016). RIM-binding protein 2 regulates release probability by fine-tuning calcium channel localization at murine hippocampal synapses. Proc Natl Acad Sci U S A..

[16] Wong King Yuen SM, Campiglio M, Tung CC, Flucher BE, Van Petegem F (2017). Structural insights into binding of STAC proteins to voltage-gated calcium channels. Proc Natl Acad Sci U S A.

[17] Iftinca MC, Altier C (2017). Stacking up Cav3.2 channels. Channels (Austin)..

[18] Bezprozvanny I, Scheller RH, Tsien RW (1995). Functional impact of syntaxin on gating of N-type and Q-type calcium channels. Nature..

[19] Zamponi GW (2003). Regulation of presynaptic calcium channels by synaptic proteins. J Pharmacol Sci..

[20] Lipscombe D, Andrade A, Allen SE (1828). Alternative splicing: functional diversity among voltage-gated calcium channels and behavioral consequences. Biochim Biophys Acta..

[21] Lipscombe D, Andrade A (2015). Calcium Channel CaValpha(1) Splice Isoforms - Tissue Specificity and Drug Action. Curr Mol Pharmacol..

[22] Li G, Wang J, Liao P, Bartels P, Zhang H, Yu D, Liang MC, Poh KK, Yu CY, Jiang F, Yong TF, Wong YP, Hu Z, Huang H, Zhang G, Galupo MJ, Bian JS, Ponniah S, Trasti SL, See K, Foo R, Hoppe UC, Herzig S, Soong TW (2017). Exclusion of alternative exon 33 of CaV1.2 calcium channels in heart is proarrhythmogenic. Proc Natl Acad Sci U S A..

[23] Wang J, Li G, Yu D, Wong YP, Yong TF, Liang MC, Liao P, Foo R, Hoppe UC, Soong TW (2018). Characterization of CaV1.2 exon 33 heterozygous knockout mice and negative correlation between Rbfox1 and CaV1.2 exon 33 expressions in human heart failure. Channels (Austin)..

[24] Powell KL, Cain SM, Ng C, Sirdesai S, David LS, Kyi M, Garcia E, Tyson JR, Reid CA, Bahlo M, Foote SJ, Snutch TP, O’Brien TJ (2009). A Cav3.2 T-type calcium channel point mutation has splice-variant-specific effects on function and segregates with seizure expression in a polygenic rat model of absence epilepsy. J Neurosci..

[25] Bell TJ, Thaler C, Castiglioni AJ, Helton TD, Lipscombe D (2004). Cell-specific alternative splicing increases calcium channel current density in the pain pathway. Neuron..

[26] Altier C, Dale CS, Kisilevsky AE, Chapman K, Castiglioni AJ, Matthews EA, Evans RM, Dickenson AH, Lipscombe D, Vergnolle N, Zamponi GW (2007). Differential role of N-type calcium channel splice isoforms in pain. J Neurosci..

[27] Jiang YQ, Andrade A, Lipscombe D (2013). Spinal morphine but not ziconotide or gabapentin analgesia is affected by alternative splicing of voltage-gated calcium channel CaV2.2 pre-mRNA. Mol Pain.

[28] Raingo J, Castiglioni AJ, Lipscombe D (2007). Alternative splicing controls G protein-dependent inhibition of N-type calcium channels in nociceptors. Nat Neurosci..

[29] Andrade A, Denome S, Jiang YQ, Marangoudakis S, Lipscombe D (2010). Opioid inhibition of N-type Ca2+ channels and spinal analgesia couple to alternative splicing. Nat Neurosci..

[30] Bean BP (1989). Neurotransmitter inhibition of neuronal calcium currents by changes in channel voltage dependence. Nature..

[31] Ikeda SR (1996). Voltage-dependent modulation of N-type calcium channels by G-protein beta gamma subunits. Nature..

[32] Herlitze S, Garcia DE, Mackie K, Hille B, Scheuer T, Catterall WA (1996). Modulation of Ca2+ channels by G-protein beta gamma subunits. Nature..

[33] Zamponi GW, Bourinet E, Nelson D, Nargeot J, Snutch TP (1997). Crosstalk between G proteins and protein kinase C mediated by the calcium channel alpha1 subunit. Nature..

[34] Abbadie C, Pan Y, Drake CT, Pasternak GW (2000). Comparative immunohistochemical distributions of carboxy terminus epitopes from the mu-opioid receptor splice variants MOR-1D, MOR-1 and MOR-1C in the mouse and rat CNS. Neuroscience..

[35] Abbadie C, Pasternak GW, Aicher SA (2001). Presynaptic localization of the carboxy-terminus epitopes of the mu opioid receptor splice variants MOR-1C and MOR-1D in the superficial laminae of the rat spinal cord. Neuroscience..

[36] Pasternak GW, Pan YX (2013). Mu opioids and their receptors: evolution of a concept. Pharmacol Rev..

[37] Xu J, Lu Z, Narayan A, Le Rouzic VP, Xu M, Hunkele A, Brown TG, Hoefer WF, Rossi GC, Rice RC, Martinez-Rivera A, Rajadhyaksha AM, Cartegni L, Bassoni DL, Pasternak GW, Pan YX (2017). Alternatively spliced mu opioid receptor C termini impact the diverse actions of morphine. J Clin Invest..

[38] Gandini MA, Souza IA, Fan J, Li K, Wang D, Zamponi GW (2019). Interactions of Rabconnectin-3 with Cav2 calcium channels. Mol Brain..

[39] Beedle AM, McRory JE, Poirot O, Doering CJ, Altier C, Barrere C, Hamid J, Nargeot J, Bourinet E, Zamponi GW (2004). Agonist-independent modulation of N-type calcium channels by ORL1 receptors. Nat Neurosci..

[40] Evans RM, You H, Hameed S, Altier C, Mezghrani A, Bourinet E, Zamponi GW (2010). Heterodimerization of ORL1 and opioid receptors and its consequences for N-type calcium channel regulation. J Biol Chem..

[41] Kisilevsky AE, Mulligan SJ, Altier C, Iftinca MC, Varela D, Tai C, Chen L, Hameed S, Hamid J, Macvicar BA, Zamponi GW (2008). D1 receptors physically interact with N-type calcium channels to regulate channel distribution and dendritic calcium entry. Neuron..

[42] Zamponi GW, Snutch TP (1998). Modulation of voltage-dependent calcium channels by G proteins. Curr Opin Neurobiol..

[43] Chan HCS, McCarthy D, Li J, Palczewski K, Yuan S (2017). Designing Safer Analgesics via mu-Opioid Receptor Pathways. Trends Pharmacol Sci..

[44] Wang HB, Zhao B, Zhong YQ, Li KC, Li ZY, Wang Q, Lu YJ, Zhang ZN, He SQ, Zheng HC, Wu SX, Hokfelt TG, Bao L, Zhang X (2010). Coexpression of delta- and mu-opioid receptors in nociceptive sensory neurons. Proc Natl Acad Sci U S A..

[45] Manglik A, Kruse AC, Kobilka TS, Thian FS, Mathiesen JM, Sunahara RK, Pardo L, Weis WI, Kobilka BK, Granier S (2012). Crystal structure of the micro-opioid receptor bound to a morphinan antagonist. Nature..

[46] Huang W, Manglik A, Venkatakrishnan AJ, Laeremans T, Feinberg EN, Sanborn AL, Kato HE, Livingston KE, Thorsen TS, Kling RC, Granier S, Gmeiner P, Husbands SM, Traynor JR, Weis WI, Steyaert J, Dror RO, Kobilka BK (2015). Structural insights into micro-opioid receptor activation. Nature..

[47] Bohn LM, Gainetdinov RR, Lin FT, Lefkowitz RJ, Caron MG (2000). Mu-opioid receptor desensitization by beta-arrestin-2 determines morphine tolerance but not dependence. Nature..

[48] Kliewer A, Schmiedel F, Sianati S, Bailey A, Bateman JT, Levitt ES, Williams JT, Christie MJ, Schulz S (2019). Phosphorylation-deficient G-protein-biased mu-opioid receptors improve analgesia and diminish tolerance but worsen opioid side effects. Nat Commun..

[49] Bourinet E, Soong TW, Stea A, Snutch TP (1996). Determinants of the G protein-dependent opioid modulation of neuronal calcium channels. Proc Natl Acad Sci U S A..

[50] Simen AA, Miller RJ (2000). Involvement of regions in domain I in the opioid receptor sensitivity of alpha1B Ca(2+) channels. Mol Pharmacol..

[51] Meza U, Adams B (1998). G-Protein-dependent facilitation of neuronal alpha1A, alpha1B, and alpha1E Ca channels. J Neurosci..

[52] Arnot MI, Stotz SC, Jarvis SE, Zamponi GW (2000). Differential modulation of N-type 1B and P/Q-type 1A calcium channels by different G protein subunit isoforms. J Physiol..

[53] Ruiz-Velasco V, Ikeda SR (2000). Multiple G-protein betagamma combinations produce voltage-dependent inhibition of N-type calcium channels in rat superior cervical ganglion neurons. J Neurosci..

[54] Berecki G, Motin L, Adams DJ (2016). Voltage-Gated R-Type Calcium Channel Inhibition via Human mu-, delta-, and kappa-opioid Receptors Is Voltage-Independently Mediated by Gbetagamma Protein Subunits. Mol Pharmacol..

