# Supplementary figures and images for: Differential regulation of Cav2.2 channel exon 37 variants by alternatively spliced μ-opioid receptors
Source: Mol Brain. 2019 Nov 27;12:98. doi: 10.1186/s13041-019-0524-6 (PMC6880636; doi:10.1186/s13041-019-0524-6)

a

Cav2.2-37a+mMOR1

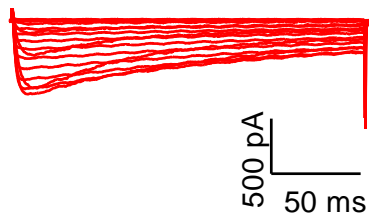

Cav2.2-37a+mMOR1O

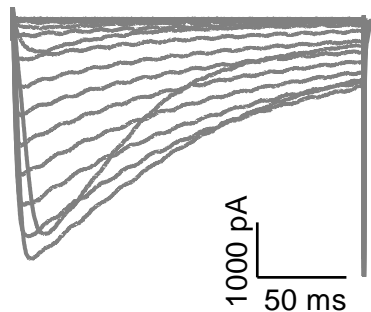

b

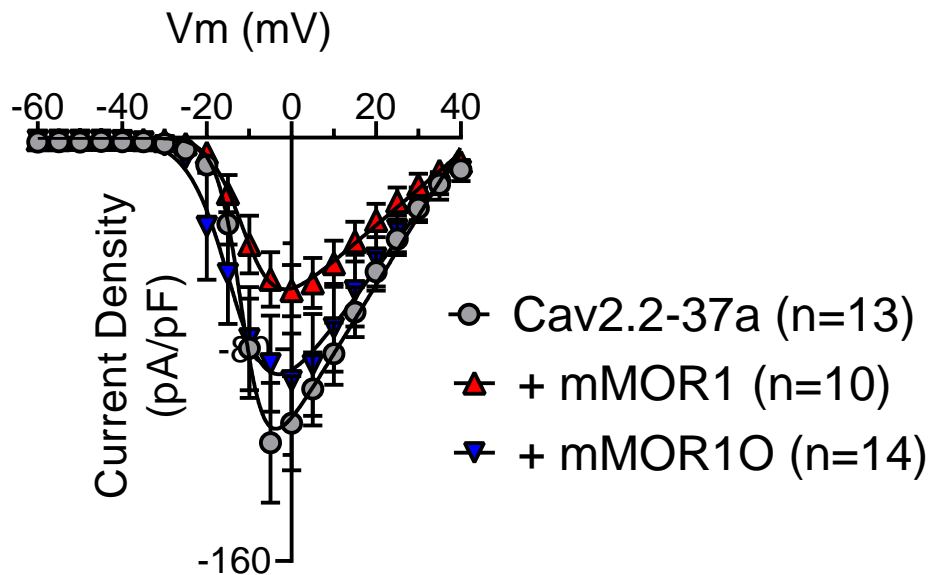

Supplement: Supplementary file 1 — Additional file 1: Figure S1. Effect of mMOR1 and mMOR1O coexpression on Cav2.2e37a channel amplitudes. a Representative whole cell current traces recorded in response to depolarizing steps from a holding potential of − 80 mV from tsA-201 cells expressing Cav2.2-37a/Cavβ1/Cavα2δ-1 in the presence of mMOR1 or mMOR1O. b Average current density-voltage relationships for cells expressing Cav2.2-37a channels with or without mMOR1 or mMOR1O. The data obtained in the absence of receptors are the same as those shown in Fig. 2b [file 13041_2019_524_MOESM1_ESM.pdf]
